# Family-Centered Prevention to Reduce Discrimination-Related Depressive Symptoms Among Black Adolescents: Secondary Analysis of a Randomized Clinical Trial

**DOI:** 10.1001/jamanetworkopen.2023.40567

**Published:** 2023-11-01

**Authors:** Steven M. Kogan, Elizabeth Kwon, Gene H. Brody, Rabeeh Azarmehr, Ava J. Reck, Tracy Anderson, Megan Sperr

**Affiliations:** 1 Department of Human Development and Family Science, University of Georgia, Athens; 2 Department of Public Health, Baylor University, Waco, Texas; 3 Center for Family Research, University of Georgia, Athens

## Abstract

**Importance:**

Racial discrimination undermines the mental health of Black adolescents. Preventive interventions that can attenuate the effects of exposure to racial discrimination are needed.

**Objective:**

To investigate whether participation in the Strong African American Families (SAAF) program moderates Black adolescents’ depressive symptoms associated with experience of racial discrimination.

**Design, Setting, and Participants:**

This secondary analysis used data from a community-based randomized clinical trial of SAAF (SAAF vs no treatment control). Participants were followed up at 10, 22, and 34 months after the baseline assessment. Assessment staff were blind to participant condition. Participants in this trial lived in 7 rural counties in Georgia. SAAF was delivered at local community centers. Eligible families had a child aged 11 to 12 years who self-identified as African American or Black. The joint influence of random assignment to SAAF and exposure to racial discrimination was investigated. Data were analyzed from September 2022 to March 2023.

**Intervention:**

SAAF is a 7-session (14 hours) family skills training intervention that occurs over 7 weeks. Small groups of caregivers and their adolescents participate in a structured curriculum targeting effective parenting behavior, adolescent self-regulation, and Black pride.

**Main Outcomes and Measures:**

The main outcome was adolescent-reported depressive symptoms, assessed at 34 months via the 20-item Center for Epidemiologic Studies Depression Scale for Children.

**Results:**

Of 825 families screened randomly from public school lists, 472 adolescents (mean [SD] age, 11.6 years; 240 [50.8%] female) were enrolled and randomized to SAAF (252 participants) or a no treatment control (220 participants). Exposure to racial discrimination at age 13 years was associated with increased depressive symptoms at age 14 years (β = 0.23; 95% CI, 0.13 to 0.34; *P* < .001). Interaction analyses indicated that the experimental condition significantly moderated the association of racial discrimination with depressive symptoms: (β = −0.27; 95% CI, −0.47 to −0.08; *P* = .005). Probing the interaction with simple slopes at ±SD revealed that for the control group, racial discrimination was significantly associated with depressive symptoms (β = 0.39; 95% CI, 0.23 to 0.54; *P* < .001), while for the SAAF group, there was no association between racial discrimination and depressive symptoms (β = 0.12; *P* = .09).

**Conclusions and Relevance:**

This randomized clinical trial found that the SAAF intervention reduced the incidence of racism-associated mental health symptoms among Black adolescents. SAAF is recommended for dissemination to health care practitioners working with rural Black adolescents.

**Trial Registration:**

ClinicalTrials.gov Identifier: NCT03590132

## Key Points

**Question:**

Can participating in a family-centered preventive intervention attenuate the association of racial discrimination with adverse mental health outcomes among Black adolescents?

**Findings:**

This secondary analysis of a randomized clinical trial including 472 Black adolescents found that adolescents randomized to the Strong African American Families (SAAF) program experienced reductions in mental health consequences associated with racial discrimination compared with adolescents in the control group.

**Meaning:**

These findings suggest that attending SAAF may reduce the mental health consequences of experiencing racial discrimination among Black adolescents.

## Teaser

This secondary analysis of a randomized clinical trial investigates whether participation in the Strong African American Families program moderates Black adolescents’ depressive symptoms associated with experience of racial discrimination.

## Introduction

Racism is a system of hierarchical categorization of social groups into races for the purpose of differential allocation of status, resources, and power in ways that privilege White individuals.^1^ Anti-Black racism in the US comprises a multilevel, multidimensional, and immersive context, a root cause of health disparities among Black individuals in the US.^2^ Building on the atrocity of chattel slavery and centuries of social, political, and economic oppression, anti-Black racism is pervasive and deeply embedded in society, systematically undermining the mental health of Black individuals over the life course and across generations.^3,4^

Life course perspectives on child development and mental health emphasize how exposure to racism during sensitive developmental periods can result in sustained shifts in developmental trajectories.^5^ Early adolescence (ages 10-14 years) is a sensitive developmental period characterized by increased responsiveness to environmental inputs, heightened reactivity to stress, and neuroplasticity.^6^ During this period, Black youth experience stressors associated with rapid biological and social changes while learning to navigate multiple, interlocking contexts (eg, family, school, neighborhood) that are shaped and influenced by racism.^7^ Individual racism from multiple perpetrators (eg, peers, teachers, shopkeepers) increases during adolescence.^8,9^ Rapid cognitive and identity developments further increase the salience and awareness of racism in one’s life.^10^ Thus, exposure to racism, as well as protective environments, during early adolescence can dramatically influence vulnerability to downstream disparities and the development of resilience to racism-related exposures.^4^

Empirical research has documented the mental health consequences of the individual-level component of racism typically referred to as *racial discrimination*, which indexes interpersonal experiences of unfair treatment based on race.^8^ Individual racism can be explicit, implicit, overt, and/or covert, manifesting as lack of respect, suspicion, devaluation, scapegoating, and dehumanization.^11,12^ Black youth self-report experiences of individual racism as young as middle childhood,^13^ and early adolescent Black children report more racism-related experiences than their peers from other ethnic and racial groups.^14,15,16,17,18^ Multiple prospective studies, including those using experience sampling methods, link racial discrimination to adolescent mental health problems.^19,20^ A recent inclusive reviews suggest that depressive symptoms are the most common mental health consequence associated with adolescents’ exposure to racial discrimination.^21^

This context underscores the need for the identification of preventive interventions that can ameliorate the influence of exposure to racial discrimination on Black adolescents’ mental health, particularly during the early adolescent years. Such attenuation of influence is referred to as *buffering*. Empirical evidence is scarce on the potential for preventive interventions to buffer the influence of racial discrimination on Black adolescents’ mental health, although recent studies have shown promise. Brody et al^22^ documented the discrimination buffering effect of 2 brief, family-centered prevention programs for older adolescents offered in community settings and evaluated in randomized trials: the Strong African American Families–Teen (SAAF-T) and the Adults in the Making (AIM) programs. Participation in the SAAF-T program attenuated the association of racial discrimination with conduct problems among Black high school students. The AIM program attenuated the association of racial discrimination with a composite assessment of depression and anxiety and a measure of conduct problems among Black 12th grade students. However, these studies do not provide evidence of the potential for family-centered intervention to buffer the influence of discrimination during the critical early adolescent period.

Two additional studies provide indirect evidence of the promise of family-centered prevention. Lei et al^23^ investigated the Protecting Strong African American Families (PROSAAF) program, a 5-session, home-based intervention for 2-parent families with an adolescent child. PROSAAF targeted effective parenting and harmonious coparenting relationships. PROSAAF was found to increase effective parenting; in a second model, effective parenting moderated the association of racial discrimination with depressive symptoms. Berkel et al^24^ examined data from a trial of the SAAF program for early adolescents. They found that SAAF promoted racial pride which, in turn interacted with discrimination and was associated with downstream psychological adjustment. Although these 2 studies^23,24^ with early adolescents suggest the potential for family-centered prevention to moderate the association of discrimination with adverse outcomes, their designs prevent strong causal inferences.

Preliminary data support the potential for family-centered prevention programs to attenuate the association of racial discrimination with depressive symptoms; however, data focusing on young adolescents is indirect and not well established. Leveraging data from a more recent prevention trial of the SAAF program^25^ (approximately 10 years after the trial analyzed in Berkel et al^24^), we conducted a secondary analysis focused on moderation of outcomes. Main effects of SAAF on alcohol use have been published elsewhere.^25^ In this secondary analysis, we hypothesized that the experimental condition (SAAF vs control) would moderate the association of discrimination with depressive symptoms. Specifically, we expected that among control group participants, discrimination at age 13 years would be associated with increases in depressive symptoms at age 14 years. In contrast, for SAAF participants, we expected that there would be no significant association between discrimination and depressive symptoms. Because emerging evidence suggests that male and female youth experience discrimination differently,^26^ we explored whether the buffering associated with SAAF varied by sex. Due to a lack of prior research, we proffered no hypotheses regarding sex differences. We also examined whether the buffering was dose dependent, requiring a specific number of SAAF sessions to manifest.

## Methods

The protocol for this randomized clinical trial was approved by the University of Georgia institutional review board. Participants provided informed consent or assent at all data collection points. We followed the Consolidated Standards of Reporting Trials (CONSORT) reporting guideline. The trial protocol and statistical analysis plan are provided in Supplement 1.

### Study Sample

**Figure 1.**
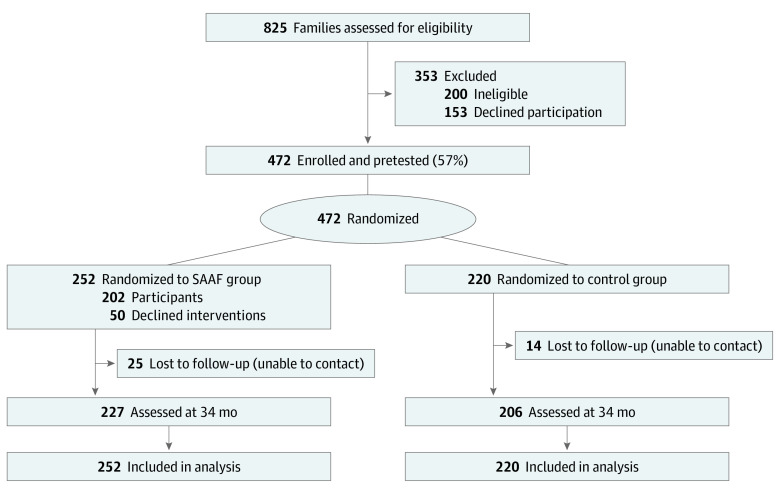
Participant Flowchart Through the Study SAAF indicates Strong African American Family program.

Hypotheses were tested with data from a randomized prevention trial of the SAAF program conducted between June 2013 and June 2019. Main effects of SAAF on adolescent alcohol use have been published elsewhere.^25^ Figure 1 presents the flowchart of participants through the trial. A sample of 472 adolescents and their primary caregivers were recruited from 7 rural counties in Georgia from June 2013 to August 2014 using lists of Black fifth-grade students in public school. Eligible families had a child aged 11 to 12 years who self-identified as African American or Black. Follow-ups occurred at 10, 22, and 34 months after baseline. In this post hoc analysis, we investigated changes in depressive symptoms from 22 months (age 13.1 years) to 34 months (age 14.1 years). Sample size was determined a priori based on power analysis for a 4-group trial that included a delayed intervention provided after 34 months, which is not analyzed in this study. Accordingly, a sample size of 115 families per group was sufficient to detect an effect of 0.13 with 0.80 power (α < .05). For this post hoc study, we conducted a Monte Carlo simulation in Mplus evaluating our power to detect an interaction effect (group assignment × discrimination) with 3 covariates. We had 80% power (α < .05) to detect a small effect (*d* < .10).

### Procedures

Families were randomly assigned to receive SAAF or no intervention (control group). Families participated in assessments at baseline and after 10, 22, and 34 months. Black research staff made home visits to collect data using audio computer-assisted self-interviews on laptop computers; they were blinded to families’ experimental assignment. Caregivers were paid $100, and adolescents were paid $40 at each assessment.

### Intervention

The SAAF intervention^25^ was designed to prevent substance use. SAAF aggregates groups of 4 to 10 families for 7 weekly, 2-hour meetings with child, parent, and family curricula.^25^ SAAF is manualized and highly structured, using prerecorded videos to time activities and enhance consistency. It was implemented by Black facilitators selected based on their interpersonal skills and experience leading community programing. Facilitators received 20 hours of training on delivering the curriculum. Teams of 3 facilitators conducted 22 SAAF groups. The possible dosage for SAAF participants was 0 to 7 sessions. Sessions were videotaped. For each group, 2 parents, 2 adolescents, and 2 family sessions were selected randomly and scored for adherence to and coverage of the prevention curriculum. Interrater reliability checks were conducted on 20% of the selected sessions (ICC = 0.80). Adherence assessments indicated that coverage of the prevention curriculum exceeded 80% of manualized activities. During this study, no harm or unintended effects were reported, and the trial ended as planned.

### Measures

#### Depressive Symptoms

Adolescents completed the 20-item Center for Epidemiologic Studies Depression Scale for Children^27^ at the 22-month (age 13 years) and 34-month (age 15 years) follow-ups. They were given a list of symptoms (eg, “I felt down and unhappy,” “It was hard to get started doing things”) and asked how often each occurred within the past week, with a response scale ranging from 0 (not at all) to 3 (a lot). Cronbach α (an index of internal consistency) was 0.87 at 22 months and 0.86 at 34 months. Items were summed, and higher levels indicate increased depressive symptoms.

#### Racial Discrimination

At 22 months, adolescents responded to a 13-item measure of racial discrimination experiences adapted from the Schedule of Racist Events^28^ and used in previous research.^29^ Adolescents were given a list of discriminatory events and were asked how often they had occurred to them in the past 6 months. Example items include, “How often have you been called a name or harassed because of your race?” and “How often did others respond to you as if they were afraid because of your race?” The response scale ranged from 1 (never) to 4 (frequently). Cronbach α at 22 months was 0.95. Higher scores represent increased exposure to racial discrimination.

### Covariates

At baseline, adolescents identified as female or male. Per past research,^30^ socioeconomic disadvantage was assessed via a risk index based on 4 dichotomous variables reported by caregivers. A score of 1 (risk factor present) or 0 (risk factor absent) was assigned to each of the following variables: family poverty based on federal guidelines, caregiver unemployment, family receipt of Temporary Assistance for Needy Families, and caregiver education level less than high school graduation or General Education Development diploma. The scores were summed to form the index, which ranged from 0 to 4.

### Statistical Analysis

Hypotheses were tested by implementing regression analysis with an interaction effect in Mplus.^31^ Missing data due to attrition (skipped survey items were negligible) from baseline to 34 months and from 22 months to 34 months were not associated with any study variables; thus, missing data were managed with full information maximum likelihood estimation.^32^ We regressed depressive symptoms at 34 months on covariates (sex, socioeconomic disadvantage, and depressive symptoms reported at 22 months), discrimination at 22 months, experimental group, and a discrimination × group interaction. A simple slopes^33^ plot was generated to probe the significant interaction effect. Significance of coefficients is presented as 95% CIs or 2-tailed tests. This study does not report on the trial’s main effect outcomes. This post hoc analysis was not preregistered; thus, study findings should be interpreted as exploratory. Data were analyzed from September 2022 to March 2023.

## Results

Staff screened 825 families and enrolled 472 families (75%). Adolescents’ mean (SD) age at the pretest assessment was 11.61 (0.62) years, and 240 (50.8%) were female. Of the sample, 252 families (53.4%) were assigned randomly to receive SAAF; 220 families (46.6%) were assigned to the control group (Figure 1). Table 1 presents sample characteristics at baseline and 22 months and differences by experimental group. At baseline, families had a mean (SD) of 2.9 (1.7) children. The primary caregivers in 418 families (88.6%) were the adolescent’s biological mothers; 25 caregivers (5.3%) were grandmothers, and 14 caregivers (3.0%) were biological fathers. The mean (SD) age among caregivers was 37.2 (8.7) years. Of the caregivers, 86 (18.2%) had less than a high school education, 125 (26.5%) had completed high school or obtained a General Education Development diploma, and the remaining 261 (55.3%) had at least some college education. Most participating families (304 families [64.4%]) had family incomes below the federal poverty threshold. Retention from baseline to 34 months was 91.7%. Attrition at 34 months was not associated with experimental condition, depressive symptoms at baseline, or baseline demographics, including adolescent sex, age, and family socioeconomic risk (eg, parental education, family poverty status). Participants attended a mean (SD) of 4.47 (2.73) sessions. Approximately 33% of participants assigned to SAAF attended all sessions, 19% attended 6 sessions, 10% attended 5 sessions, 18% attended between 1 and 4 sessions, and 20% attended no sessions.

**Table 1.** Sample Characteristics by Experimental Group

Variables	Mean (SD)		
	Full Sample (n = 472)	SAAF (n = 252)	Control (n = 220)
SES disadvantage at baseline^a^	1.77 (1.15)	1.80 (1.17)	1.74 (1.12)
Sex	0.49 (0.50)	0.50 (0.50)	0.48 (0.50)
Depressive symptoms^b^			
Baseline	16.76 (10.75)	16.83 (10.31)	16.67 (11.26)
22 mo	15.53 (11.47)	15.75 (11.62)	15.29 (11.32)
Discrimination at 22 mo^c^	20.13 (9.18)	20.25 (9.61)	20 (8.71)

Abbreviations: SAAF, Strong African American Families; SES, socioeconomic status.

^a^ Range, 0 to 4; higher score indicates more disadvantage.

^b^ Range, 0 to 60; higher score indicates more depressive symptoms.

^c^ Range, 13 to 52; higher score indicates more experiences of racial discrimination.

**Figure 2.**
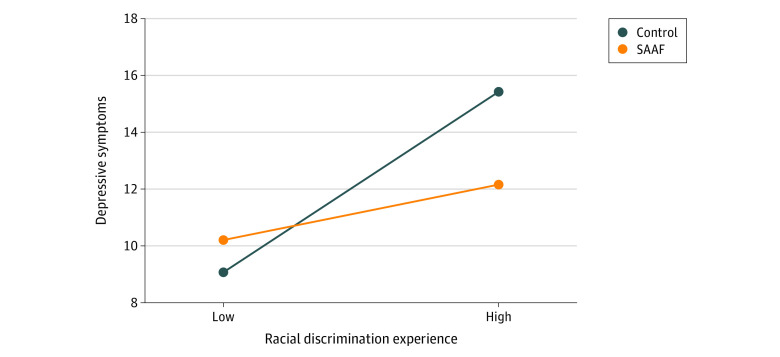
Simple Slopes of the Moderating Effect of the Strong African American Family (SAAF) Intervention on the Association Between Racial Discrimination and Depressive Symptoms Low discrimination, indicates 1 SD below the mean; high discrimination, 1 SD above the mean.

Regression models are presented in Table 2. Per model 2, for every unit increase in discrimination experienced at 22 months, depressive symptoms at 34 months increased by 0.23 (β = 0.23; 95% CI, 0.13 to 0.34; *P* < .001); the main effect of group on depressive symptoms was not significant. The interaction term (model 3) was significantly associated with depressive symptoms at 34 months (β = −0.27; 95% CI, −0.47 to −0.08; *P* = .01). To probe the interaction effect, Figure 2 presents a simple slopes diagram. Consistent with study hypotheses, for the control group, racial discrimination was significantly associated with increases in depressive symptoms (β = 0.39; 95% CI, 0.23 to 0.54; *P* < .001); for the SAAF group, there was no significant association (β = 0.12; *P* = .09). For each 1-SD increase in discrimination experience, depressive symptoms were higher in the control group (unadjusted mean, 13.09; adjusted mean, 15.60) compared with the SAAF group (unadjusted mean, 11.8; adjusted mean, 11.81). Participant sex did not affect the association of group and racial discrimination with depression (β = 0.01; 95% CI, −0.34 to 0.06; *P* = .52).

**Table 2.** Hierarchical Regression Analysis for Depressive Symptoms at 34 Months

Variable	Model 1^a^		Model 2^b^		Model 3^c^	
	β (SE) [95% CI]	*P* value	β (SE), 95% CI	*P* value	β (SE), 95% CI	*P* value
Sex^d^	0.48 (0.04) [−3.21 to 0.50]	.15	−1.91 (0.93) [−3.75 to −0.08	.04	−1.90 (0.91) [−3.70 to −0.10]	.03
SES disadvantage at 22 mo	−1.35 (0.94) [−0.68 to 2.07]	.32	0.59 (0.68) [−0.74 to 1.94	.38	0.55 (0.67) [−0.76 to 1.88]	.40
Depressive symptoms at 22 mo	0.69 (0.70) [0.40 to 0.57]	<.001	0.42 (0.04) [0.34 to 0.51]	<.001	0.42 (0.04) [0.34 to 0.51]	<.001
Group^e^	NA	NA	−0.05 (0.93) [−1.84 to 1.83]	>.99	−0.46 (0.45) [−1.35 to 0.42]	.30
Discrimination at 22 mo	NA	NA	0.23 (0.05) [0.13 to 0.34]	<.001	0.39 (0.07) [0.23 to 0.54]	<.001
Group × discrimination at 22 mo	NA	NA	NA	NA	−0.27 (0.10) [−0.47 to −0.08]	.01

Abbreviations: NA, not applicable; SES, socioeconomic status.

^a^ Model 1 included covariates.

^b^ Model 2 included covariates and direct effects.

^c^ Model 3 included covariates, direct effects, and interaction.

^d^ Sex was dichotomized as female, 1; male, 2.

^e^ Group is the SAAF intervention.

We further probed dose effects (0-7 sessions) with the SAAF group subsample. Dose significantly interacted with discrimination to reduce the association with depressive symptoms (β = −0.27; 95% CI, −0.47 to −0.08). To probe the interaction effect, we used the Johnson Neyman technique^34^ (eFigure in Supplement 2). Results indicated that at doses of fewer than 5 SAAF sessions, the association of discrimination on depressive symptoms remained significant. However, for youth receiving 5 or more sessions, buffering was not significant (ie, there was no significant association between racial discrimination and depressive symptoms).

## Discussion

In this secondary analysis of a randomized clinical trial, we hypothesized that participation in the SAAF program would attenuate the association of racial discrimination at age 13 years with increases in depressive symptoms at age 14 years. Secondary analysis of SAAF trial data confirmed study hypotheses. For adolescents who were not assigned to SAAF, racial discrimination was associated with significant increases depressive symptoms; however, for adolescents whose families were assigned to SAAF, there was no significant association of experiences of racial discrimination with depressive symptoms. This moderation was observed using an intent-to-treat design and accounting for family socioeconomic disadvantage and youth sex. These findings highlight the need for preventive interventions that can buffer adolescents from the consequences of discrimination.

Considerable evidence from clinical and animal model research underscores how social relationships in general, and family relationships in particular, play critical roles in regulating physiological and neurocognitive responses to stress and trauma.^35,36,37,38,39^ Individuals who experience their family environments as socially supportive and consistent respond to stress with less biological reactivity,^35^ including quicker stabilization of cortisol levels, heart rate, and blood pressure.^36^ Among Black youth, parenting processes associated with nurturance, consistent discipline, and racial socialization can attenuate the influence of racial discrimination on youths’ well-being.^18,23,40,41,42^ Thus, family-centered preventive interventions during early adolescence have the potential to impact addictive behavior trajectories among Black adolescents.

Study findings are consistent with recent research on the buffering influence of family-centered interventions among older adolescents^22^ and indirect evidence that the processes targeted in prevention programs can attenuate the impact of racial discrimination.^23,24^ These studies, in conjunction with accumulating evidence from cohort studies,^18,41,43^ suggest that prevention programs targeting aspects of racial identity, racial socialization processes, and parenting behavior may, to some extent, mitigate the mental health effects associated with racial discrimination. These processes appear to increase positive coping in the aftermath of discrimination,^44^ and prevent adolescents’ internalization of toxic messages regarding racial inferiority.^45^

Study findings may also be interpreted from the perspective of racial discrimination as a moderator of intervention effects. This suggests that in the context of racial discrimination, SAAF’s efficacy may be amplified. It is plausible that in the presence of elevated discrimination, SAAF is particularly salient and engaging for adolescents, augmenting the effects of the curriculum content. This is consistent with research demonstrating that prevention effects may be amplified by baseline risk exposure.^46,47^

### Limitations

This study has some limitations. First, this is a post hoc, rather than preplanned, analysis of trial data. Because the sample consisted of Black adolescents from rural areas of Georgia, the generalizability of study findings to other settings is unknown. The use of a no treatment control group (rather than an attention control) does not permit ruling out nonspecific intervention factors (such as social support) as potential explanations for study findings. Due to the study’s focus on individual level racial discrimination, the potential for SAAF to buffer the effects of structural and institutional forms of racism that we did not measure is unknown. Future research examining processes and interventions that buffer youth mental health from these forms of racism is needed.^7^ These limitations notwithstanding, this study’s experimental design is noteworthy. It provides experimental evidence of the potential for family-centered intervention to mitigate the effects of racial discrimination addressing a critical public health need.

## Conclusions

The findings of this secondary analysis of a randomized clinical trial of the SAAF program suggest that family-centered prevention programming may reduce the depressive symptoms associated with racial discrimination among Black youths. These findings support the widespread dissemination of SAAF as part of a strategy for ameliorating disparities that Black families experience.

## Notes

Supplement 1.
Trial Protocol and Statistical Analysis Plan

Supplement 2. **eFigure.** Johnson Neyman Plot of the Interaction Effect of Discrimination With Dose on Depressive Symptoms

Supplement 3.
Data Sharing Statement
