# Supplementary material for: Family-Centered Prevention to Reduce Discrimination-Related Depressive Symptoms Among Black Adolescents: Secondary Analysis of a Randomized Clinical Trial
Source: JAMA Netw Open. 2023 Nov 1;6(11):e2340567. doi: 10.1001/jamanetworkopen.2023.40567 (PMC10620615; doi:10.1001/jamanetworkopen.2023.40567)
Supplement: Supplement 1. — Trial Protocol and Statistical Analysis Plan [file jamanetwopen-e2340567-s001.pdf]

Trial Protocol

Participants

A random sample of 460 African American youth in 5th grade and their primary caregivers will be recruited for the proposed study. Families will be sampled from 8 Georgia counties. We selected nonmetropolitan counties (population density <100 persons per square mile) with large proportions of African American residents (>25%) that represent the range of non-urban environments in the South in which African American live [1]. These areas are demographically and economically similar to those in which SAAF and SAAF–T were originally tested. Table 1 presents county demographic characteristics. In past trials, we examined the possibility that county heterogeneity in these indicators affected intervention response: no effects were detected [2]

Table 1. Demographic characteristics of the counties to be included in the sample (AA = African American)

County	AA (%)	Single Parent	Median AA Family Income ($k)	AA <HS (%)
Baldwin	43	48	26.2	38
Burke	50	51	17	45
Emanuel	32	50	15.6	47
Green	38	50	21.4	46
Laurens	35	49	20.0	43
MacDuffie	38	50	20.1	50
Screven	43	44	24.9	47
Warren	56	50	19.7	56

Using data from the Department of Education , we estimated a pool of no less than 800 eligible households from which to sample 460 families (54.5%). Based on recruitment rates in our prior prevention trials (65%-90% of eligible household), an ample number of families will be available for us to reach our sampling goals.

Methodological Overview.

Figure 4 presents an overview of the study design.

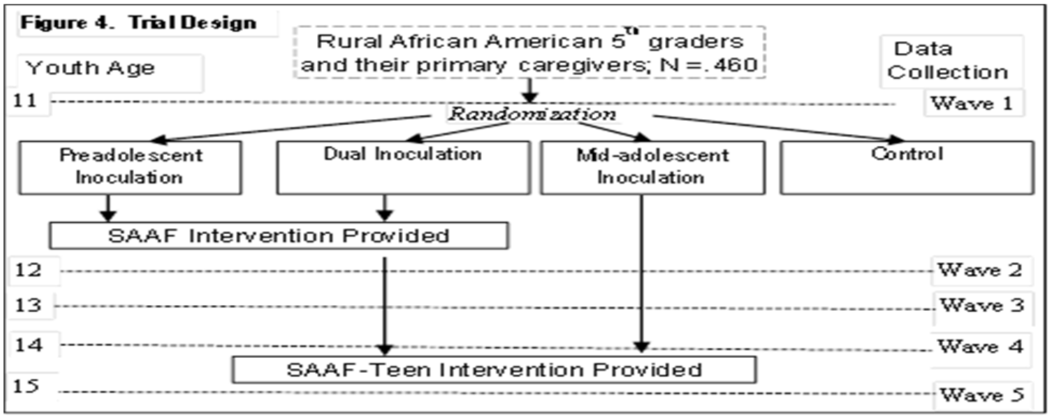

Sampling frame and eligibility.

Consistent with our prior prevention research protocols [3, 4], youth and their primary caregivers will be recruited in random order from lists of students in public schools in the targeted counties. Very few (<2%) rural African American youth in these areas attend private schools; thus, lists of public-school students approximate a population frame. Eligibility criteria for youth include age of 10-11 years and identification by the family and school as African American. Primary caregivers are not required to identify as African American. Because we are not currently working with families of early adolescents in the areas listed in Table 1, few families will already be taking part in one of our projects. In the unlikely event that a family is already participating in a CFR project, that family will be ineligible for recruitment. Siblings and stepsiblings who both are in the 5th grade will be excluded, because the inclusion of more than one participant from the same family introduces confounds that make interpretation difficult.

Random assignment and contamination.

After pretest, families will be assigned randomly to one of four experimental groups: (a) *preadolescent inoculation*, (b) *mid-adolescent inoculation*, (c) *dual inoculation* control, and (d) control. Although the efficacy of SAAF and SAAF–Teen have been established, inclusion of a

control condition provides a baseline from which to compare normative alcohol use behavior in the population to both single- and dual-inoculation groups; it is also essential for comparing the single inoculations' economic efficiency. Preadolescent inoculation families will be assigned to receive SAAF at age 11 with no further intervention. Mid-adolescent inoculation families will receive SAAF–Teen at age 14. The dual-inoculation group will be assigned to receive both SAAF at age 11 and SAAF–Teen at age 14. The control condition involves no intervention contact. Participants will be notified if they are selected to participate in SAAF shortly after pretest. No participants will know if they are to receive SAAF–Teen until several months prior to its implementation. For each intervention implementation, families who are not attending a program receive written information by mail regarding adolescent development and alcohol use. Sending these materials is intended to maintain investment in the project for families who are not attending an intervention. No alcohol use effects are expected based on receiving written materials. All families will be followed across the course of the proposed study and assessed regardless of the number of intervention sessions they attend (intent-to-treat design). Information will be collected from family members about their participation in family enrichment programs offered in the school or community. The potential effects of participation in other programs (contamination) will be examined and statistically controlled in data analyses, as Lang et al. [5] described.

Repeated assessments and attrition.

Families will participate in 5 annual assessments. Wave 1, the baseline assessment will occur when youth are age 11 and approximately 3 months prior to implementation of SAAF. Wave 4 will occur just prior to the implementation of the SAAF–Teen. The final assessment, Wave 5, occurs approximately 10 months after implementation of SAAF—Teen. In previous

basic research and prevention trials conducted at CFR, 5-year retention rates ranged from 88% to 92% [6, 7]. We plan conservatively for a 13% attrition rate, ensuring that 400 participants (87% of 460) will complete the trial. Figure 5 presents the project timeline.

Figure 5. Project Time Line

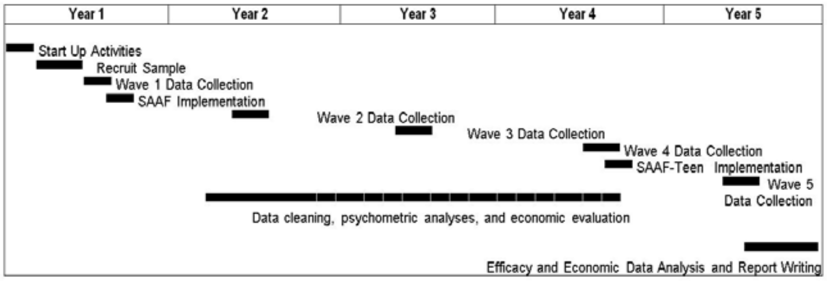

Family Recruitment and Retention

The recruitment procedures will be similar to protocols used in our previous prevention trials with rural African American families. These protocols rely on community liaisons (CLs), residents of the counties from which families are sampled; they serve as contacts between CFR and the communities. They are selected on the basis of their positive reputations and extensive social contacts in their communities. The CLs work with the recruitment coordinator to enroll families, then remain in contact with participating families and track changes in the addresses and phone numbers of families who move.

Family recruitment will be conducted as follows:

(a) <u>Sampling lists.</u> As in the investigators' past and ongoing studies [8, 9], research staff work with school staff to identify eligible youth and develop a list from which to sample. We have excellent relationships with schools in the counties listed in Table 1 (see Section 14 for support letters from school officials).

(b) <u>Introductory letter.</u> Caregivers of African American youth receive a letter introducing CFR, emphasizing its focus on health and resilience in African American communities, and informing caregivers that researchers are interested in screening families in the community for participation in a research project. Families are advised to expect a phone call from a local

representative (the CL).

(c) Phone screening. CLs follow up letters with phone screenings to primary caregivers to determine potential eligibility. CLs call families from participant lists in random order to ensure diverse representation of available families. If a family is potentially eligible, a screening/enrollment home visit is scheduled.

(d) Home visit. At the home visits, CLs confirm eligibility, describe the project in detail, and answer any questions the family members may have. Family members are informed that all project participants are expected to participate in annual data collection home visits over the next 5 years. In addition to these home visits, some families, selected by "chance," will be asked to participate in a prevention program when their youth enter 6th grade, a similar program 3 years later, or both programs. Families must agree to attend if they are selected for either of the programs. If parents and youth agree, a pretest assessment visit is scheduled. Informed consent/assent is obtained at pretest.

Monetary incentives.

At each data collection, parents will receive $80 and target youth will receive $20. Focus group feedback from rural African Americans indicated that these amounts are appropriate compensation for a 1–2-hour data collection visit and acknowledge that the researchers consider their time to be valuable. We explicitly explored in focus groups and post-data-collection debriefings the potential for this level of financial incentive to be overtly coercive for families who are economically distressed. Neither community members nor the UGA IRB consider this level of compensation to be coercive [10].

Retention of trial participants.

CFR retention protocols have yielded high retention rates with African American

families followed for time periods ranging from 3 to 14 years [11]. At each data collection, family members provide contact information for three or more individuals who will always know their whereabouts. We gather email addresses, cell phone numbers, and Internet social networking addresses for participants and their primary contacts. Following each data collection visit, project staff contact participants to inquire about the quality of the experience, confirm contact information, and answer questions. Between data collections, we phone participants every 3 months to update contact information. Participants also receive a project newsletter that includes a stamped address correction form, as well as birthday cards and holiday cards. Experienced retention staff work with participants' contacts, CLs, and commercial databases such as NetDetective to locate lost participants. Participants' 5-year rates of mobility outside of Georgia are low (~3%). At each follow-up, we are prepared to send a data collection team to participants' homes throughout the region to collect data from families who have moved. For participants who leave the region, we collect data by phone or online; our research teams have experience with both modes of data collection.

Preventive Intervention Implementation Procedures

Procedures for recruiting and training intervention facilitators, insuring curriculum fidelity, and engaging participants are based on our experiences in previous trials with rural African American families including the previous evaluation of SAAF and SAAF–T [3, 12, 13].

Intervention facilitators.

SAAF/SAAF–T intervention facilitators are African Americans with a minimum education level of high school graduation. They are selected based on information from interviews and references that attest to their communication skills, engaging personalities, and ability to implement a structured program with fidelity. Facilitators will receive 30 hours of

training on the implementation of each program. Training includes curriculum activities, guided practice in delivering and pacing curriculum segments, and facilitator self-care. They will work from detailed manuals that describe all facets of program delivery. Didactic material, role-playing exercises, and modeling will be used to teach the protocol for each session. To ensure ongoing quality control and fidelity, facilitators will meet with the Prevention Coordinator weekly.

Intervention engagement and re-engagement.

Families who are randomized to an intervention condition will receive an introductory visit from their assigned facilitator prior to the first group-based session. This visit is designed to familiarize parents with the program, answer questions about the intervention, and increase the family's comfort with the facilitator. During the visit, family members will view a promotional video describing the program. They will be asked about their weekly schedules, and sessions will be planned to accommodate the schedules of as many families as possible. To facilitate attendance, during the first half hour of each intervention session, a meal will be served to family members attending the session and any siblings of the target youth who accompany their parents. Assistance with transportation and on-site childcare will be provided on request. CLs will call all participants before the program begins and prior to each weekly session to encourage their involvement. Reminder postcards will be sent from the CFR each week, highlighting the focus of the upcoming meeting. A CL will call participants who miss a meeting to remind them of the next meeting and address any barriers to attendance. A $25 honorarium will be provided to each family for each session attended, to defray any expenses associated with attendance. *Feasibility of re-engagement***.** Engagement of the majority of families assigned to receive SAAF–Teen at mid-adolescence is made feasible by low rates of geographic mobility among rural African

American families in the targeted counties, and the routine retention and engagement protocols implemented by our experienced staff. Prior to implementation of SAAF–Teen in project year 4, families will have participated in ongoing data collection activities that involve a minimum of three home visits. At each home visit, families are reminded that they may be selected to participate in an intervention in the future. Data collection visits as well as the protocols discussed in Section C.4 (birthday cards, newsletters, etc.) strengthen families' investment in the project. As with SAAF participants in project year 1, all families assigned to receive SAAF–Teen will experience the same protocols designed to achieve high rates of participation, including a program information visit, childcare and transportation assistance, a meal at each session, and a $25 honorarium. Over 90% of eligible families of high school students agreed to participate in the SAAF–Teen program during the initial prevention trials [4].

Prevention programs and program fidelity.

Content and structure of the SAAF and SAAF–Teen programs are described in Section A. Complete facilitator manuals are provided in Appendix A (SAAF) and Appendix B (SAAF–Teen). Standardized procedures insure program fidelity. The intervention manuals include a detailed format to be used for each session. Each facilitator will be provided with materials designed to support correct execution of the session protocol, including an outline of each session, a checklist of the materials necessary for each activity, the specific theme of each task, and forms for in-session notes. Presentation of targeted processes on videotapes helps to ensure fidelity. Each session is audiotaped. Each team of facilitators is assigned an intervention supervisor, an expert trainer with a graduate degree in applied behavioral science, who will review session audiotapes with facilitators and provide weekly supervision of the intervention process. To assess program fidelity, session audiotapes will be scored for protocol adherence

using instruments validated in the SAAF and SAAF–Teen trials. Two judges will score 20% of the sessions to assess interrater reliability (κ). In the past studies, mean coverage of the prevention curriculum components was >90% [4, 14].

Data Collection Procedures

At each of the five assessments, one 2-hour home visit will be made to each family for data collection. To minimize cultural bias, African American community members, many of whom have worked at CFR for years, will serve as field researchers. Field researchers will receive 16 hours of training and supervised practice that will address obtaining informed consent, building rapport, and helping participants to use the computer-based survey system. Field researchers will meet weekly with the Project Coordinator to discuss any problems that arise. Each year, a 5-hour refresher training course will be conducted. Data will be gathered via audio computer-assisted self-interviewing (ACASI) technology. Compared with face-to-face interviews and written surveys, ACASI elicits less social desirability bias and more accurate reports of socially sensitive issues [15, 16]. The user-friendly program guides respondents through the survey; those with low literacy skills are assisted through audio enhancements. ACASI was used successfully in prior trials. *Consent/Assent.* Parents will consent to their own and their youths' participation at each data collection visit. Youth will assent to participation. Consent/assent forms are provided in Appendix C.

Measures

We selected measures validated with rural African American youth and parents in the SAAF and SAAF–Teen trials. Space limitations preclude full descriptions of the measures indexing SAAF and SAAF–Teen targeted protective processes; see Appendix D for copies of all measures.

Demographics (Waves 1-5).

Primary caregivers will report their yearly income, per capita income, employment status, duration of unemployment during the past 2 years, hours worked per week, education level, numbers of children and adults in the household, and relations of all household members to one another.

Alcohol and other drug use (Waves 1-5).

The substance use assessment is designed to address the multidimensional nature of use. Items were selected from three well-validated measures for their sensitivity to intervention effects in our previous research at several levels of substance use. At the initiation level, group differences in the proportion of participants who transition from nonuse to use during the study can be detected. At the escalation level, group differences in the proportion of participants who increase their frequency/intensity of use during the study can be assessed. At the problem use level, group differences in specific substance-related behaviors and problems can be indexed. The basic items in the measure are those included in the Monitoring the Future study, which assess lifetime, annual, and 30-day prevalence and frequency of cigarette smoking, alcohol use, binge drinking, and marijuana use. For the purposes of the proposed study, a 3-month assessment will be added. A checklist of other drugs includes lifetime use of LSD/acid, Ecstasy, cocaine, heroin, other opiates, amphetamines, non-prescribed tranquilizers, and inhalants. Problems associated with substance use will be indexed using items from the Minnesota Student Survey for high school students [17] and the Rutgers Alcohol Problems Index [18]. These scales include items assessing dependence (e.g., had to use more to get same effect, tried to cut down but couldn't) and negative consequences of use (e.g., hurt relationships with friends or family, interfered with other activities, resulted in legal problems, missed school or work).

SAAF Targeted Protective Practices (Waves 1-3).

The SAAF Intervention-Targeted Processes instrument assesses parenting and individual youth processes on which SAAF sessions focus. The battery includes parallel versions of items that permit youth and parents to report the frequency of parenting practices and parent-youth relationship dynamics we termed *regulated/communicative parenting.* Items include those from well-established measures of relationships climate, disciplinary practices, and racial socialization processes that were adapted for African American families with the assistance of focus groups [14], as well as items specifically written for the SAAF trial and validated with those data. Subscales, confirmed via factor analysis, include involved vigilant parenting, racial socialization, and clear expectations about alcohol use and other risky behavior. Items assess behavior in the past 6 weeks with responses that range from 1 (*never*) to 5 (*always*). Cronbach's alphas for the subscales ranged from .70-.92 in our prior prevention research and were sensitive to intervention participation [14]. Items assessing youths' *intrapersonal protective processes* targeted in SAAF include the following subscales: *behavioral and emotional self-regulation, racial identity, prototypes of alcohol-using peers, and resistance efficacy*. Youth report their intrapersonal protective processes and parents report their observations of youths' self-control. As with the family items, these scales were developed from existing items, adaptations derived from focus group feedback, and specific items written for the SAAF trial. They were confirmed with factor analyses, and Cronbach's alphas ranged from .68-.90 [2, 14, 19, 20].

SAAF–Teen Targeted Protective Practices (Waves 4 and 5).

The SAAF–Teen Intervention-Targeted Protective Processes instrument (see Appendix D for all items) assesses parenting and individual youth processes on which SAAF–Teen sessions focus. As with the SAAF instruments, the SAAF–Teen battery includes parallel versions

of items that permit youth and parents to report the frequency of developmentally appropriate parenting behaviors that we termed *competence-promoting parenting.* Many items represent developmentally appropriate items, adapted via wording changes and behavioral emphases, of items used in SAAF (limit setting, consistent discipline) as well as new items that address unique intervention targets such as parents' provision of support and coaching for dealing with discrimination and for planning academic and vocational careers. Additional items address parental involvement in youths' educational pursuits and problem-solving interactions that foster youths' sense of autonomy and competence. Youth subscales emphasize goal setting and planning; self-regulatory processes and skills for coping with stress, particularly racial discrimination; and decision-making processes in peer contexts. Subscale reliability ranged from .70-.92 in our prior prevention research, and the scales were sensitive to intervention participation [4].

Data Analysis

Data management, exploratory data analysis, psychometrics, and experimental considerations.

Data will be organized and cleaned following quality control procedures that involve analyses of outliers and unexpected or inappropriate missing-data patterns. Next, items' distributions and constructs' psychometric properties will be examined. We will establish equivalence among experimental conditions with ANOVAs and chi-square tests on baseline levels of alcohol use, preadolescent protective processes, and demographic variables. Any departures from equivalence will be controlled in subsequent analyses. All primary analyses will be intent-to-treat.

Differential retention and intervention participation.

Our prior experience and the aforementioned retention and intervention engagement procedures support our expectation that attrition will pose minimal threats to the internal validity of the study. We will, however, investigate the possibility of differential retention or participation by experimental condition. When data are missing consistent with a “missing at random” (MAR) assumption [21], we will test hypotheses with models that handle missing data using full maximum likelihood (FIML) methods in which all available data are included [22]. Non-ignorable missing data will be managed with procedures that explicitly model “missingness mechanisms” to develop unbiased causal parameters [23, 24]. For example, although unlikely, if experimental groups report differential attrition or levels of participation in assigned interventions, complier average causal effect (CACE) methods [25, 26] can be used to estimate accurately the causal effect of dual inoculation versus other groups. CACE models use a mixture modeling framework to develop groups based on compliance status. Comparisons of treatment groups among the “compliers” provide unbiased estimates of causal effects.

Figure 6: Aim 1

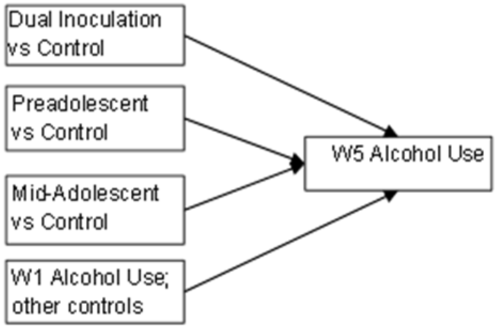

Aim 1.

Mean differences in alcohol use by experimental group at wave 5 (~10 months after SAAF–T implementation) will be tested with structural equation models (SEM) as presented in Figure 6. These models are roughly equivalent to ANCOVA, but the SEM software permits FIML-based estimation of parameters as well as options for forming latent alcohol use constructs and greater precision in specifying and testing model parameters. We will investigate main effects on onset, frequency of use, binge use, and alcohol use problems as well as aggregate alcohol risk indices validated in prior trials [2, 27] to establish mean differences in use as a

consequence of treatment assignment. We will model outcomes with logistic (e.g., onset analyses), continuous, Poisson, or Negative Binomial distributions as appropriate. Ancillary analyses will examine intervention effects on marijuana and nicotine use. We also will consider group differences in alcohol use trajectories using latent growth curve models that permit evaluation of differences in the rates of change (including nonlinear trajectories) in alcohol use. We have used these techniques extensively in past research [2, 7, 28].

Power.

For experimental comparisons among four groups, a sample size of 100-115 families per group is sufficient to detect an effect of .13 with .80 power (alpha $< .05$) and a power of .90 for an effect as small as .15. For growth curve analyses ($n > 100$ per group), we used algorithms [29] to estimate the smallest intervention effect size we can detect with 80% power for changes in the slope parameter, assuming linear growth over four time points. With measurement reliability of 60%, we can achieve 80% power when the ES is 0.1 at posttest and ES is 0.2 at long-term follow-up. This suggests that we will have sufficient power to detect small to moderate intervention effects over time.

Aim 2.

This aim examines pathways through which different inoculation strategies affect distal (W5) alcohol use. Analyses in Aim 1 serve as a baseline for examining mediation and the subsequent decomposition of intermediate effects. SEM provides a parsimonious tool

Figure 7. General model for SEM of intervening processes

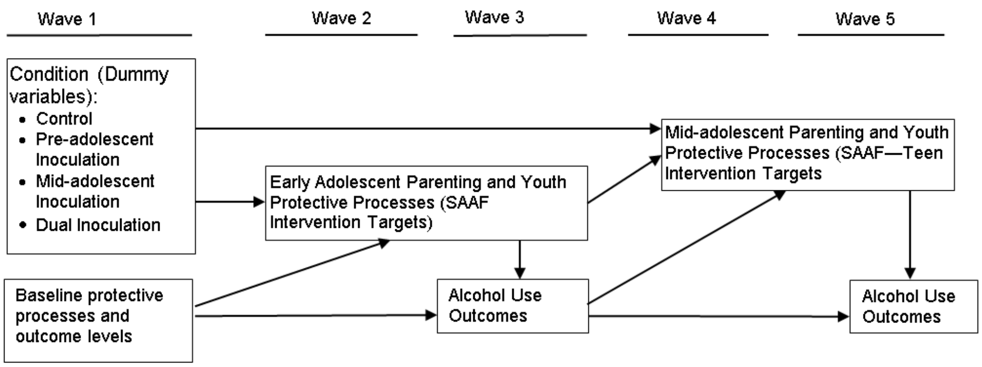

that enables the analyst to identify indirect pathways of influence between intervention assignment and high school alcohol use outcomes. The potential mediators include SAAF-targeted protective processes, SAAF–Teen-targeted protective processes, and preadolescent (Wave 3) alcohol use. Figure 7 provides a general specification for intervening variable models. Baseline or pretest levels of distal and proximal processes will be controlled in all analyses. For example, in analyses of SAAF–T intervention-targeted processes as a mediator, pretest (Wave 4) levels of these processes will be controlled when testing for posttest (Wave 5) changes. Because multiple protective processes are targeted in each program, we will approach their operationalization with diverse methods. First, we will create scores that index the number of protective processes that a youth or family evinces using established cutoff scores. Such indices account for the numbers of protective factors present rather than variability in any particular factor. We have used this approach previously, and it is sensitive to intervention effects [12]. We have also had success in forming latent constructs from sets of interrelated intervention-targeted mediators [14]. In these analyses, we will confirm the protective processes' underlying dimensionality and examine invariance in the protective factors' measurement model across time. We will test uncorrelated protective processes in separate models. Significance of mediational effect tests will be assessed with bootstrapping methods per MacKinnon [30].

Statistical power. With Monte Carlo simulation [31] (n = 100 per group), we evaluated power to detect the significance of path coefficients that vary in their "true" magnitude, holding the other parameters in the model constant at values estimated from previous research [2, 32]. For the paths included in Aim 2, power ranged from .7 to .9 for detecting path coefficients ranging from .2 to .4 ($p < .05$). This suggests that we will have the power to detect small and medium effect sizes among model paths.

Aim 3. Incremental CEA of alcohol use initiation prevented, and level of alcohol abuse deterred.

We will test the hypotheses that a dual inoculation is cost-effective in deterring alcohol use outcomes relative to other experimental groups. Separate CEAs will be conducted for each different outcome including alcohol use initiation, use escalation, alcohol problems, and health-related quality of life from two perspectives: society and intervention provider. The time frame for the intervention is 5 or 7 weeks. The analytic horizon for the CEA, that is, the period over which costs and outcomes will be measured, is 4 years. Costs and outcomes will be converted to present-day values using a 3% discount rate [33], with 2%-10% used in a sensitivity analysis [34]. Future costs and outcomes will be discounted back to the time when the intervention first occurred. The intent-to-treat analyses of intervention effects discussed in Aim 1 will be used for estimating outcome effects.

Best-practice guidelines [33, 35] prescribe calculating an incremental cost-effectiveness ratio (ICER) when comparing the cost-effectiveness of one approach (dual inoculation) with others (control, preadolescent inoculation, mid-adolescent inoculation). The ICER represents the incremental costs (from the aforementioned cost analysis) divided by the incremental effectiveness. The result is a measure of the net costs of the intervention for each one-unit gain in the outcome measure: for example, the cost per case of alcohol problems prevented.

Per best practice [36, 37], we will account for uncertainty in the ICER with cost-effectiveness acceptability curves (CEACs), derived from the joint density of the individual-level costs and effects of the intervention of interest. A CEAC is generated by estimating net benefits of an intervention:

$$NB = \lambda(TotalHealthOutcomes\Pr evented_B - TotalHealthOutcomes\Pr evented_A)$$
$$-[(InterventionCost_B - HealthCostAverted_B) - (InterventionCost_A - HealthCostAverted_A)]$$

The key features of NB are that it no longer includes a ratio, that one can calculate the *p* (NB > 0) at alternative values of λ, and that these values can be plotted, generating the cost-effectiveness acceptability curve. The CEAC thus represents the proportion of the density at which the intervention is cost-effective for a range of values of λ. The λ, which is unknown, represents the policy maker's varying degrees of tolerance for different resource allocation decisions, and the CEAC provides the necessary information for an informed decision under uncertainty. Because we do not know the threshold for λ below which decision makers would choose to implement the intervention based on the CEA result and above which they would choose not to implement, we will construct CEACs for varying levels of λ. To provide an additional level of sensitivity analysis, parametric estimation of the joint densities will be estimated indirectly via parametric bootstrapping and Monte Carlo simulation using the individual-level data [38]. These methods allow for the uncertainty of the ICER to be represented on the cost-effectiveness plane along with a visual representation of the ICER confidence intervals.

References

1. Probst J, Samuels M, Jespersen K, Willer K, Swann R, McDuffie J. Minorities in rural America: An overview of population characteristics. Columbia, SC: University of South Carolina, Norman J. *Arnold School of Public Health*. 2002.
2. Brody GH, Murry VM, Kogan SM, Gerrard M, Gibbons FX, Molgaard V, et al. The Strong African American Families Program: a cluster-randomized prevention trial of long-term effects and a mediational model. *Journal of consulting and clinical psycholog*y. 2006;74(2):356.
3. Mrazek PJ, Haggerty RJ. Reducing risks for mental disorders: Frontiers for preventive intervention research: *National Academy Press*; 1994.
4. Kogan SM, Brody GH, Molgaard VK, Grange CM, Oliver DA, Anderson TN, et al. The Strong African American Families–Teen trial: Rationale, design, engagement processes, and family-specific effects. *Prevention Science*. 2012;13:206-17.
5. Lang DL, DiClemente RJ, Hardin JW, Crosby RA, Salazar LF, Hertzberg VS. Threats of cross-contamination on effects of a sexual risk reduction intervention: Fact or fiction. *Prevention Science*. 2009;10:270-5.
6. Brody GH, Kim S, Murry VM, Brown AC. Protective longitudinal paths linking child competence to behavioral problems among African American siblings. *Child Development*. 2004;75(2):455-67.
7. Brody GH, Chen YF, Murry VM, Ge X, Simons RL, Gibbons FX, et al. Perceived discrimination and the adjustment of African American youths: A five-year longitudinal analysis with contextual moderation effects. *Child development*. 2006;77(5):1170-89.
8. Nash SG, McQueen A, Bray JH. Pathways to adolescent alcohol use: Family environment, peer influence, and parental expectations. *Journal of adolescent health*. 2005;37(1):19-28.

9. Brody GH, Forehand R. Prospective associations among family form, family processes, and adolescents' alcohol and drug use. *Behaviour research and therapy*. 1993;31(6):587-93.

10. Murry VM, Brody GH. Partnering with community stakeholders: Engaging rural African American families in basic research and the Strong African American Families preventive intervention program. *Journal of Marital and Family Therapy*. 2004;30(3):271-83.

11. Simons RL, Simons LG, Lei MK, Landor AM. Relational schemas, hostile romantic relationships, and beliefs about marriage among young African American adults. *Journal of social and personal relationships*. 2012;29(1):77-101.

12. Brody GH, Kogan SM, Chen Y-f, Murry VM. Long-term effects of the strong African American families program on youths' conduct problems. *Journal of Adolescent Health*. 2008;43(5):474-81.

13. Rohrbach LA, Grana R, Sussman S, Valente TW. Type II translation: transporting prevention interventions from research to real-world settings. *Evaluation & the health professions*. 2006;29(3):302-33.

14. Brody GH, Murry VM, Gerrard M, Gibbons FX, Molgaard V, McNair L, et al. The strong African American families program: Translating research into prevention programming. *Child development.* 2004;75(3):900-17.

15. Cohen MA. The monetary value of saving a high-risk youth. *Journal of quantitative criminology.* 1998;14:5-33.

16. Spoth RL, Guyll M, Day SX. Universal family-focused interventions in alcohol-use disorder prevention: cost-effectiveness and cost-benefit analyses of two interventions. *Journal of Studies on Alcohol*. 2002;63(2):219-28.

17. Harrison PA, Fulkerson JA, Beebe TJ. DSM-IV substance use disorder criteria for adolescents: A critical examination based on a statewide school survey. *American Journal of Psychiatry.* 1998;155(4):486-92.

18. White HR, Labouvie EW. Towards the assessment of adolescent problem drinking. *Journal of studies on alcohol*. 1989;50(1):30-7.

19. Brody GH, Murry VM, Gerrard M, Gibbons FX, McNair L, Brown AC, et al. The strong African American families program: prevention of youths' high-risk behavior and a test of a model of change. *Journal of family Psychology*. 2006;20(1):1.

20. Brody GH, McBride Murry V, McNair L, Chen YF, Gibbons FX, Gerrard M, et al. Linking changes in parenting to parent–child relationship quality and youth self-control: The Strong African American Families Program. *Journal of Research on Adolescence*. 2005;15(1):47-69.

21. Little RJ, Rubin DB. Statistical analysis with missing data: John Wiley & Sons; 2019.

22. Arbuckle JL, Marcoulides GA, Schumacker RE. Full information estimation in the presence of incomplete data. A*dvanced structural equation modeling: Issues and techniques*. 1996;243:277.

23. Birmingham J, Fitzmaurice GM. A pattern-mixture model for longitudinal binary responses with nonignorable nonresponse. *Biometrics*. 2002;58(4):989-96.

24. Demirtas H, Schafer JL. ON THE PERFORMANCE OF RANDOM-COEFFICIENT PATTERN-MIXTURE MODELS FOR NONIGNORABLE ATTRITION.

25. Chen H, Geng Z, Zhou XH. Identifiability and estimation of causal effects in randomized trials with noncompliance and completely nonignorable missing data. *Biometrics*. 2009;65(3):675-82.

26. Connell AM. Employing complier average causal effect analytic methods to examine effects of randomized encouragement trials. *The American journal of drug and alcohol abuse*. 2009;35(4):253-9.

27. Brody GH, Chen Y-f, Kogan SM, Yu T, Molgaard VK, DiClemente RJ, et al. Family-centered program deters substance use, conduct problems, and depressive symptoms in black adolescents. *Pediatrics*. 2012;129(1):108-15.

28. Kogan SM, Yu T, Brody GH, Chen Y-f, DiClemente RJ, Wingood GM, et al. Integrating condom skills into family-centered prevention: Efficacy of the Strong African American Families–Teen program. *Journal of Adolescent Health*. 2012;51(2):164-70.

29. Brown CH, Kellam SG, Kaupert S, Muthén BO, Wang W, Muthén LK, et al. Partnerships for the design, conduct, and analysis of effectiveness, and implementation research: experiences of the

prevention science and methodology group. Administration and Policy in Mental Health and Mental *Health Services Research*. 2012;39:301-16.

30. MacKinnon DP, Lockwood CM, Hoffman JM, West SG, Sheets V. A comparison of methods to test mediation and other intervening variable effects. *Psychological methods*. 2002;7(1):83.

31. Jöreskog KG, Sörbom D. LISREL 8: User's reference guide: Scientific Software International; 1996.

32. Beach SR, Hurt TR, Fincham FD, Franklin KJ, McNair LM, Stanley SM. Enhancing marital enrichment through spirituality: Efficacy data for prayer focused relationship enhancement. *Psychology of Religion and Spirituality*. 2011;3(3):201.

33. Neumann PJ, Sanders GD, Russell LB, Siegel JE, Ganiats TG. Cost-effectiveness in health and medicine: Oxford University Press; 2016.

34. Boardman AE, Laurin C, Moore MA, Vining AR. A cost-benefit analysis of the privatization of Canadian national railway. *Canadian Public Policy*. 2009;35(1):59-83.

35. Haddix AC, Teutsch SM, Corso PS. Prevention effectiveness: a guide to decision analysis and economic evaluation: Oxford University Press; 2002.

36. Fenwick E, Claxton K, Sculpher M. Representing uncertainty: the role of cost-effectiveness acceptability curves. *Health economics*. 2001;10(8):779-87.

37. Fenwick E, O'Brien BJ, Briggs A. Cost-effectiveness acceptability curves–facts, fallacies and frequently asked questions. *Health economics*. 2004;13(5):405-15.

38. Briggs A, Fenn P. Confidence intervals or surfaces? Uncertainty on the cost-effectiveness plane. *Health economics*. 1998;7(8):723-40.
