# Supplementary material for: Family-Centered Prevention to Reduce Discrimination-Related Depressive Symptoms Among Black Adolescents: Secondary Analysis of a Randomized Clinical Trial
Source: JAMA Netw Open. 2023 Nov 1;6(11):e2340567. doi: 10.1001/jamanetworkopen.2023.40567 (PMC10620615; doi:10.1001/jamanetworkopen.2023.40567)
Supplement: Supplement 2. — eFigure. Johnson Neyman Plot of the Interaction Effect of Discrimination With Dose on Depressive Symptoms [file jamanetwopen-e2340567-s002.pdf]

Supplemental Online Content

Kogan SM, Kwon E, Brody GH, et al. Family-centered prevention to reduce discrimination-related depressive symptoms among Black adolescents: secondary analysis of a randomized clinical trial. *JAMA Netw Open*. 2023;6(11):e2340567.
doi:10.1001/jamanetworkopen.2023.40567

eFigure. Johnson Neyman Plot of the Interaction Effect of Discrimination With Dose on Depressive Symptoms

This supplemental material has been provided by the authors to give readers additional information about their work.

© 2023 Kogan SM et al. *JAMA Network Open.*

eFigure. Johnson Neyman plot of the interaction effect of Discrimination with Dose on depressive symptoms

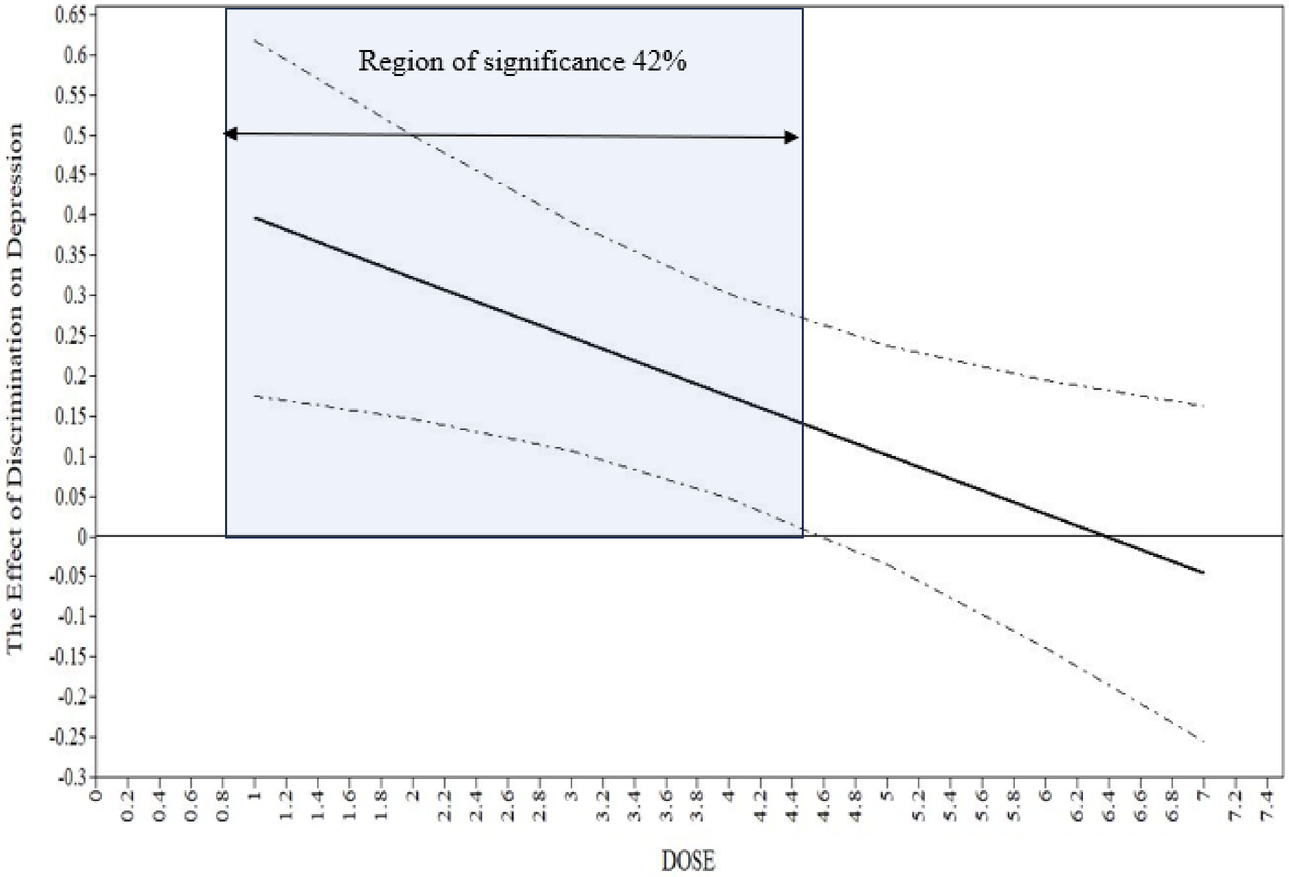

Dose, (0-7 SAAF intervention sessions); The x-axis represents Dose Effect as the moderator. The y-axis represents the unstandardized coefficient of the effects of Discrimination on Depressive symptoms. The gray shaded areas reflect regions of significance for the moderating effects.

© 2023 Kogan SM et al. *JAMA Network Open.*
