# Supplementary material for: Family-Centered Prevention to Reduce Discrimination-Related Depressive Symptoms Among Black Adolescents: Secondary Analysis of a Randomized Clinical Trial
Source: JAMA Netw Open. 2023 Nov 1;6(11):e2340567. doi: 10.1001/jamanetworkopen.2023.40567 (PMC10620615; doi:10.1001/jamanetworkopen.2023.40567)
Supplement: Supplement 3. — Data Sharing Statement [file jamanetwopen-e2340567-s003.pdf]

Data Sharing Statement

Kogan. Family-Centered Prevention to Reduce Discrimination-Related Depressive Symptoms Among Black Adolescents. *JAMA Netw Open*. Published November 01, 2023. doi:10.1001/jamanetworkopen.2023.40567

Data
Data available: No

Additional Information
Explanation for why data not available: The identified data will be shared upon request by the primary author to certified researchers under a data-sharing agreement.
